# Regulation of atypical MAP kinases ERK3 and ERK4 by the phosphatase DUSP2

**DOI:** 10.1038/srep43471

**Published:** 2017-03-02

**Authors:** Maria Perander, Rania Al-Mahdi, Thomas C. Jensen, Jennifer A. L. Nunn, Hanne Kildalsen, Bjarne Johansen, Mads Gabrielsen, Stephen M. Keyse, Ole-Morten Seternes

**Affiliations:** 1 Department of Medical Biology, UiT The Arctic University of Norway, N-9037 Tromsø, Norway; 2 Department of Pharmacy UiT The Arctic University of Norway, N-9037 Tromsø, Norway; 3 Stress Response Laboratory, Division of Cancer Research, Jacqui Wood Cancer Centre, James Arrot Drive, Ninewells Hospital and Medical School, Dundee DD1 9SY, UK

## Abstract

The atypical MAP kinases ERK3 and ERK4 are activated by phosphorylation of a serine residue lying within the activation loop signature sequence S-E-G. However, the regulation of ERK3 and ERK4 phosphorylation and activity is poorly understood. Here we report that the inducible nuclear dual-specificity MAP kinase phosphatase (MKP) DUSP2, a known regulator of the ERK and p38 MAPKs, is unique amongst the MKP family in being able to bind to both ERK3 and ERK4. This interaction is mediated by a conserved common docking (CD) domain within the carboxyl-terminal domains of ERK3 and ERK4 and the conserved kinase interaction motif (KIM) located within the non-catalytic amino terminus of DUSP2. This interaction is direct and results in the dephosphorylation of ERK3 and ERK4 and the stabilization of DUSP2. In the case of ERK4 its ability to stabilize DUSP2 requires its kinase activity. Finally, we demonstrate that expression of DUSP2 inhibits ERK3 and ERK4-mediated activation of its downstream substrate MK5. We conclude that the activity of DUSP2 is not restricted to the classical MAPK pathways and that DUSP2 can also regulate the atypical ERK3/4-MK5 signalling pathway in mammalian cells.

ERK3 and ERK4 are members of the so-called “atypical” family of mitogen activated protein kinases (MAPKs), which also includes ERK7/8 and Nemo Like Kinase (NLK)^1^. One major difference between the ERK3 and ERK4 and classical MAP kinases such as ERK1/2, p38 isoforms, and c-Jun N-terminal kinases (JNKs), is the substitution of the conserved T-X-Y motif within the activation loop by a single phospho-acceptor residue within an S-E-G motif^2^,^3^. Phosphorylation of the serine residue within this motif is instrumental for the ability of both ERK3 and ERK4 to interact with and activate MAPK-activated protein kinase 5 (MK5 or PRAK), the only well-characterized physiological substrate of these atypical MAPKs. The regulation of both ERK3 and ERK4 activity is poorly understood, as is the physiological context in which activation can occur. Recently, members of the class I p21-activated kinases (PAKS) were shown to mediate robust phosphorylation of the S-E-G motif resulting in enzymatic activation of both ERK3 and ERK4 and downstream activation of MK5/PRAK^4^,^5^. Interestingly, phosphorylation of the S-E-G motif in ERK4 is also substantially increased on binding to MK5 but this does not require MK5 kinase activity, indicating that MK5 either facilitates the activity of an ERK4 kinase or prevents the dephosphorylation and inactivation of ERK4^6^.

The classical MAPKs interact with both upstream activators, regulatory proteins and substrates through conserved clusters of acidic amino acids, which constitute the so called “common docking” (CD) domain^7^. In contrast, we recently reported that the interaction of both ERK3 and ERK4 with MK5 is mediated by a novel (FRIEDE) MAPK binding motif found in the C-terminal extension of both kinases^8^. However, the observation that both ERK3 and ERK4 contain a conserved consensus sequence motif indicative of a functional CD domain strongly suggests that these kinases may utilize this binding site to interact with as yet unidentified regulatory proteins.

Prominent amongst candidate regulatory proteins, which interact with MAPKs via the CD domain, are a family of ten dual-specificity MAPK phosphatases (MKPs or DUSPs)^9^. These phosphatases act in direct opposition to the upstream dual specificity MAPK kinase (MKK or MEK) by dephosphorylating both the tyrosine and threonine residues within the T-X-Y motif, thus inactivating the MAPK. MKPs interact with the cognate CD domain in MAPKs via a conserved kinase interaction motif (KIM), which is located within the amino-terminal non-catalytic domain of the phosphatase. It is thought that variations in the modular sequence of this KIM combined with the presence of either nuclear localisation signals (NLS) or nuclear export signals (NES) determine the differential substrate specificity and subcellular localisation of the MKPs^10^.

Here we have used a yeast 2-hybrid assay to screen all ten members of the mammalian MKP family for the ability to interact with ERK3 and 4 and find that these kinases specifically interact with DUSP2, a phosphatase previously identified as a regulator of signalling through both the ERK and p38 MAPK pathways^11^,^12^,^13^. The interaction of DUSP2 with both ERK3 and ERK4 in yeast and mammalian cells required a functional KIM and was abolished by mutation of the CD domain in both ERK3 and ERK4. Furthermore, the formation of a complex between ERK4 and DUSP2 led to an increase in the stability of DUSP2, which was at least in part dependent on the catalytic activity of ERK4. Importantly, the expression of wild-type DUSP2 led to efficient dephosphorylation of the phospho-acceptor site within the S-E-G motif of both ERK3 and 4 *in vivo*. Finally, we demonstrate that DUSP2 inhibits ERK3 and ERK4 mediated activation of MK5. We conclude that the activity of DUSP2 is not restricted to the classical MAPK pathways and that uniquely amongst this family of enzymes DUSP2 is also a physiological regulator of the atypical ERK3/4 MAPKs in mammalian cells.

## Results

### The Inducible MAP kinase phosphatase encoded by DUSP2 interacts with the atypical MAP kinases ERK3 and ERK4

**Figure 1:**
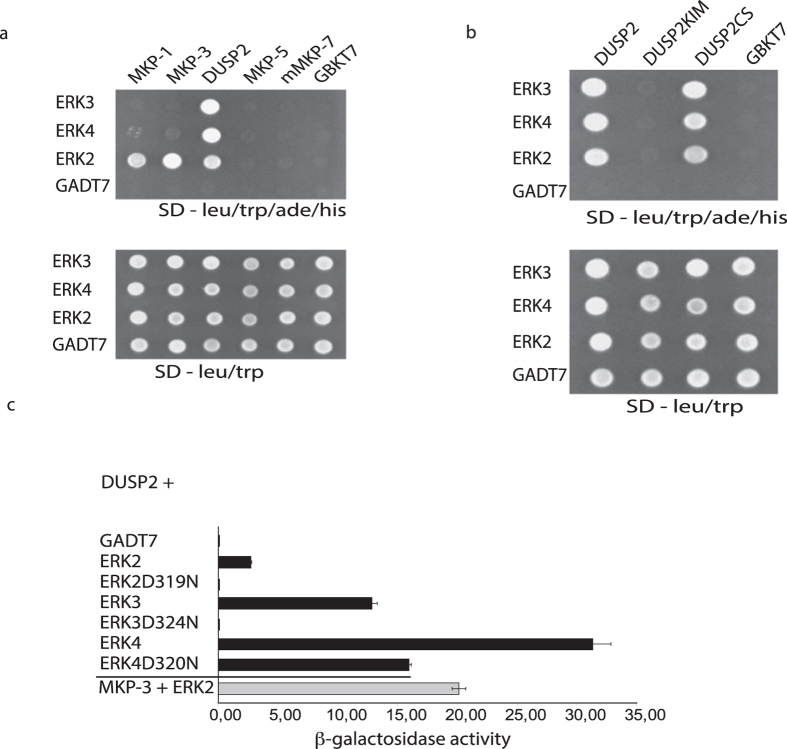
Yeast 2-hybrid assays reveal a specific interaction between DUSP2 and the atypical MAPKs ERK3 and ERK4. (**a)** ERK3 and ERK4 interact specifically with DUSP2 in yeast. Expression vectors (pGADT7) encoding GAL4 activation domain fusion proteins of ERK3, ERK4 or ERK2 were transformed into the yeast strain Y187. Selected transformants were mated with transformants of yeast strain PJ69-2A containing expression vectors (pGBKT7) encoding different members of the MAP kinase phosphatase family fused to the GAL4 DNA-binding domain. Yeast diploids expressing both DNA-binding domain and activation domain fusions were selected on synthetic drop out (SD) medium deficient for leucine and tryptophan. Protein-protein interactions were assessed by growth on SD medium lacking leucine, tryptophan, alanine and histidine. (**b**) DUSP2 interacts with ERK3 and ERK4 in a KIM-dependent manner independently of its catalytic activity. Yeast strain Y187 expressing GAL4 activation domain fusion proteins of ERK3, ERK4 or ERK2 were mated with PJ69-2A expressing wild-type DUSP2, a DUSP2 mutant lacking a functional KIM (DUSP2KIM) or a catalytically inactive DUSP2 mutant (DUSP2CS). Protein-protein interactions were determined as described in a. (**c**) ERK3 and ERK4 interact with DUSP2 via their CD domains. Semi-quantitative analysis of yeast two-hybrid interactions based on the level of induction of the b-galactosidase reporter gene were performed for the interactions between DUSP2 and wild-type ERK3, ERK4 and ERK2, or versions of the respective MAP kinases carrying a point mutation within the CD domain. The interaction between the ERK-specific phosphatase MKP-3 and ERK2 is included as a positive control. Assays were performed in triplicate and means are presented with associated errors.

As a first step in determining if the atypical MAPKs ERK3 and ERK4 might be subject to regulatory dephosphorylation we tested a number of human dual-specificity MAPK phosphatases for their ability to interact with these atypical MAPKs using the yeast 2-hybrid assay. These assays revealed that, in addition to its well-characterized ability to interact with ERK2, DUSP2 was also able to interact with both ERK3 and ERK4. In contrast, the inducible nuclear MKP DUSP1/MKP-1, which can interact with ERK1/2, p38α and JNK1^14^, did not bind to either ERK3 or ERK4. Nor did the cytoplasmic ERK1/2-specific MKP DUSP6/MKP-3 or either of the p38/JNK-specific MKPs DUSP10/MKP-5 or DUSP16/MKP-7 (Fig. 1a). In additional yeast 2-hybrid assays, no interactions were detected between ERK3 or ERK4 and the other 5 members of the MKP family^15^,^16^ and data not shown. The ability of DUSP2 to interact with ERK2, ERK3 and ERK4 was absolutely dependent on the integrity of the kinase interaction motif (KIM) located within the amino-terminal non-catalytic domain of the phosphatase as mutation of conserved arginine residues within this region abolished the interaction. In contrast, binding to ERK2, ERK3, and ERK4 do not require DUSP2 catalytic activity, as a mutation of the active site cysteine to serine (C257S), did not affect its ability to interact with all three MAPKs (Fig. 1b). The requirement for the KIM in DUSP2 suggests that these interactions are mediated via the conserved “common docking” (CD) domain of these MAPKs. This is confirmed, as mutants in which this docking site is disrupted by mutation of a conserved aspartic acid residue (D319N, in ERK2, D324N in ERK3 and D320N in ERK4) abolishes the ability of DUSP2 to bind to ERK2 and ERK3 and greatly reduces the interaction of DUSP2 with ERK4 (Fig. 1c).

**Figure 2:**
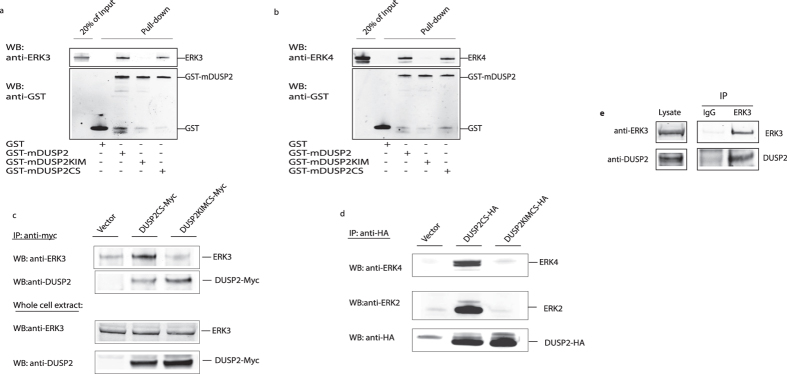
DUSP2 interacts with ERK3 and ERK4 in a KIM-dependent manner both *in vitro* and *in vivo.* Two micrograms of either ERK3 (**a**) or ERK4 (**b**) were incubated with 2 μg of GST, GST-mDUSP2, GST-mDUSP2KIM or GST-mDUSPC and glutathione agarose. Following GST pulldown, bound ERK3 or ERK4 was detected by western-blotting using an anti-ERK3 (**a**) or anti-ERK4 (**b**) antibody, respectively. GST and GST-fusions were visualized using an anti-GST antibody. (**c**) NCI-H1299 cells were transfected with either an empty expression vector or the plasmids encoding a myc-tagged catalytically inactive mutant of DUSP2 (DUSP2CS-Myc) or catalytically inactive DUSP2 in which the KIM motif was also mutated (DUSP2CSKIM-Myc) respectively. Twenty-four hours after transfection, cells were lysed and myc-tagged DUSP2 was immunoprecipitated from the lysate using an anti-Myc monoclonal antibody. Co-immunoprecipitated endogenous ERK3 was detected by Western-blotting using the anti-ERK3 (clone 4C11) antibody (upper panel) Immunoprecipitated DUSP2 was detected by Western-blotting using a sheep anti-DUSP2 antibody (second panel). The expression of endogenous ERK3 and overexpressed myc-DUSP2 in the lysates were verified by Western-blotting using a monoclonal anti-ERK3 (clone 4C11) antibody and polyclonal anti-DUSP2 antibody respectively (third and fourth panel). (**d**) HEK-293 cells were transfected with either empty expression vector or the plasmids DUSP2CS-HA or DUSP2CSKIM-HA respectively. Twenty-four hours after transfection cells were lysed and HA-tagged DUSP2 was immunoprecipitated from the lysate using an anti-HA monoclonal antibody. Co-immunoprecipitated endogenous ERK4 and ERK2 were detected by Western-blotting using the polyclonal anti-ERK4 antibody (upper panel) and polyclonal ERK2 antibody (second panel). Immunoprecipitated DUSP2 was detected by Western-blotting using a monoclonal anti-HA antibody (third panel). (**e**) Jurkat T-cells were stimulated with PMA and anti-CD3 antibody for 3 hours in presence of the proteosome inhibitor MG132. Endogenous ERK3 was immunoprecipitated from the cleared lysate using 2 ug goat polyclonal anti-ERK3 antibody (ERK3), a preimmune sheep IgG antibody (IgG) was used as a non-specific control. The immunoprecipitates were probed for ERK3 using a monoclonal anti-ERK3 antibody (clone 4C11, upper panel) and for DUSP2, (lower panel) using the anti-DUSP2 antibody. The presence of ERK3 and DUSP2 in the lysate was verified by western-blot of the lysate using the same antibodies. Unprocessed original scans of the blots are shown in Supplementary Fig. 1

To show that these interactions are direct, GST pull-down assays were performed using purified recombinant ERK3 and ERK4 and a fusion between GST and the murine homologue of DUSP2. Both wild-type and a catalytically inactive (C261S) mutant of DUSP2 clearly interacted directly with both ERK3 and ERK4. In contrast, the KIM mutant of DUSP2 was completely unable to interact with either ERK3 or ERK4 (Fig. 2a and b).

ERK3 and ERK4 also formed complexes with wild-type and catalytically inactive DUSP2 when co-expressed in in HeLa cells (Fig. 2c and d). The inactive mutant (DUSP2CS) is used here to act as a potential substrate trap^17^. In both cases this interaction was lost when the KIM motif was mutated. We could also detect interaction between endogenous ERK3 and endogenous DUSP2 in Jurkat T-cells stimulated with PMA and OKT3 (Fig. 2e) indicating that the interactions are physiologically relevant. We can conclude that, unlike other dual-specificity MKPs, DUSP2 is able to interact with the atypical MAPKs ERK3 and ERK4 both *in vitro* and in mammalian cells.

### The interaction between DUSP2 and either ERK3 or ERK4 does not result in catalytic activation of DUSP2

**Figure 3:**
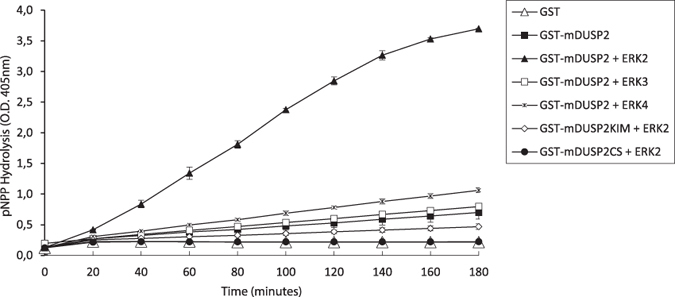
The catalytic activity of DUSP2 is increased by ERK2 but not by either ERK3 or ERK4 *in vitro*. Time dependent hydrolysis of *p*-Nitrophenylphosphate (*p*-NPP) by either recombinant wild-type murine DUSP2, catalytically inactive DUSP2 (DUSP2CS), or DUSP2 with a KIM mutation (DUSP2KIM) either in the absence or presence of recombinant ERK2, ERK3, or ERK4 as indicated was monitored by measuring the change in the optical density (O.D.) at 405 nm. Results from two independent experiments are shown. Standard deviations are calculated from the mean values of triplicate samples.

Several dual-specificity MKPs exhibit catalytic activation on binding to their cognate MAPK substrates *in vitro*. Thus the activities of DUSP6/MKP-3 and DUSP1/MKP-1 towards the chromogenic substrate *para*-nitrophenyl phosphate (*p*-NPP) are significantly increased on addition of either recombinant ERK2 (DUSP6/MKP-3) or either ERK2, p38α, or JNK1 (DUSP1/MKP-1)^14^,^18^. This activation is due to allosteric rearrangement of residues within the active site of these enzymes on substrate binding and, in particular, on a conformational change, which places a conserved general acid residue in an optimal position for catalysis^19^. Catalytic activation has also been observed when DUSP2 is incubated in the presence of recombinant ERK2^20^. We tested the ability of recombinant ERK2, ERK3, and ERK4 to activate recombinant murine DUSP2 *in vitro*. As previously reported, addition of ERK2 resulted in a significant increase in the rate of *p*-NPP hydrolysis by DUSP2 and this did not occur when the KIM motif was mutated (Fig. 3). In contrast, addition of recombinant ERK3 or ERK4 did not lead to any significant increase in DUSP2 activity, indicating that although both ERK3 and ERK4 can bind to DUSP2, this binding does not result in catalytic activation of the phosphatase.

### DUSP2 can dephosphorylate both ERK3 and ERK4 when expressed in mammalian cells

**Figure 4:**
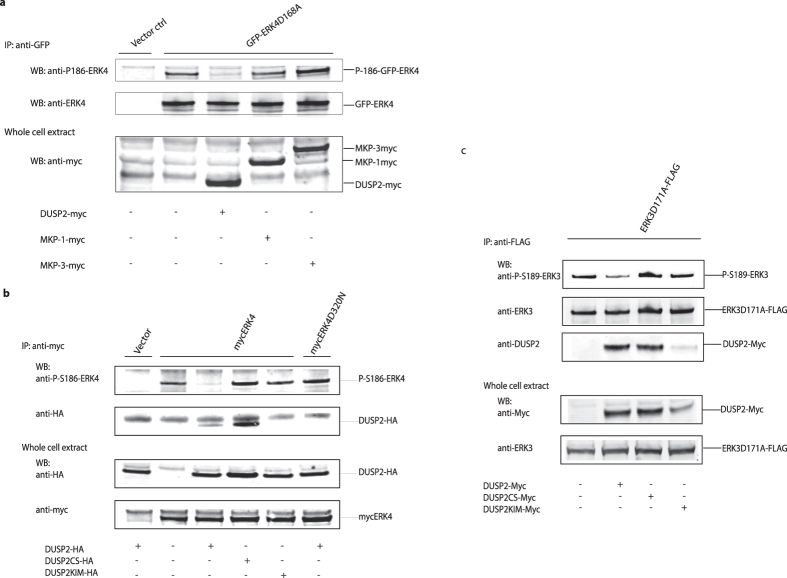
DUSP2 dephosphorylates Serine 189 and 186 in the activation loop of ERK3 and ERK4 respectively *in vivo*. (**a**) HeLa cells were co-transfected with expression vectors encoding a kinase-dead mutant of ERK4 fused to green fluorescent protein (GFP-ERK4D168A) and either myc-tagged DUSP2, MKP-1, or MKP-3. After 24 h, whole cell extracts were prepared and GFP-ERK4D168A was immunoprecipitated using an anti-GFP antibody. The phosphorylation status of Serine 186 within the activation loop in immunoprecipitated ERK4 D168A was then analysed by Western blotting using an anti-phospho S186 antibody. The levels of GFP-ERK4D168A in immunoprecipitates and of the three MKPs in whole cell extracts were analysed by Western blotting using antibodies against ERK4 and myc, respectively. (**b**) HeLa cells were co-transfected with vectors encoding either myc-tagged ERK4 or ERK4D320N together with either HA-tagged DUSP2, DUSP2KIM, or DUSP2CS. After 24 h, whole cell extracts were prepared and either ERK4 or ERK4D320N were immunoprecipitated using an anti-myc antibody. The phosphorylation status of Serine 186 was analysed as described in A. Co-immunoprecipitated DUSP2 was visualized using an anti-HA antibody. The levels of overexpressed ERK4- and DUSP2 proteins were analysed by Western blotting of whole cell extracts using anti-myc and anti–HA antibodies, respectively. (**c**) NCI-H1299 cells were co-transfected with expression vectors encoding a Flag-tagged kinase-dead mutant of ERK3 (ERK3D171A) and either an empty expression vector or plasmids encoding wild-type DUSP2, a catalytically inactive DUSP2 mutant (DUSP2CS) or a kinase interaction motif-deficient mutant (DUSP2KIM) respectively. After 24 h, cell extracts were prepared and ERK3 was immunoprecipitated using M2-FLAG conjugated agarose. The Phosphorylation status of Serine 189 within the activation loop of immunoprecipitated ERK3 was analysed by Western-blotting using a specific anti-phospho Serine 189 ERK3 antibody. Levels of ERK3 protein in the input lysates and immunoprecipitates were analysed by Western-blotting using an anti-FLAG antibody. The expression of myc-tagged DUSP2 proteins in cell lysates and immunoprecipitates were analysed by Western-blotting using an anti-myc or anti-DUSP2 antibody respectively. All experiments were performed 3 times and representative images are shown. Unprocessed original scans of the blots are shown in Supplementary Fig. 1

To test the ability of DUSP2 to dephosphorylate ERK4 in mammalian cells, we co-expressed either wild-type DUSP2, DUSP1/MKP-1, or DUSP6/MKP-3 with a GFP fusion of a kinase deficient mutant of ERK4 (D168A) in HeLa cells. Kinase dead mutants of ERK4 (and ERK3, see below) were used in these experiments to prevent autophosphorylation of the activation loop reversing the cellular activity of the DUSP2 phosphatase during the immunoprecipitation procedure, which is carried out in buffers containing the phosphatase inhibitor sodium orthovanadate. ERK4 was then immunoprecipitated from cell lysates using an anti-GFP antibody and Western blots were performed using an antibody that specifically recognizes ERK4 when phosphorylated at S186 within the S-E-G motif. Expression of DUSP2 clearly caused a significant reduction in the phosphorylation of S186, while the expression of either DUSP1/MKP-1 or DUSP6/MKP-3 had no effect on this modification (Fig. 4a). The DUSP2-mediated dephosphorylation of ERK4 was strictly dependent on the intrinsic phosphatase activity as strong interaction, but no dephosphorylation, was seen upon coexpression of the catalytically inactive (S261) mutant of DUSP2 (DUSP2 CS) (Fig. 4b). ERK4 with a mutated common docking (CD) domain (ERK4D320N) was not dephosphorylated upon DUSP2 coexpression and DUSP2 with a mutated KIM domain did not dephosphorylate wild-type ERK4 (Fig. 4b). This strongly indicates that direct interaction between the two proteins is necessary for DUSP2-mediated ERK4 dephosphorylation. The same was observed when DUSP2 was coexpressed in HeLa cells with a kinase-inactive mutant of ERK3(D171A) (Fig. 4c). In contrast, neither a catalytically deficient nor a KIM mutant of DUSP2 was able to dephosphorylate ERK3. We conclude that DUSP2 is able to dephosphorylate both ERK3 and ERK4 when expressed in mammalian cells.

### The FRIEDE motif within ERK3 and ERK4 is not required for binding to DUSP2

**Figure 5:**
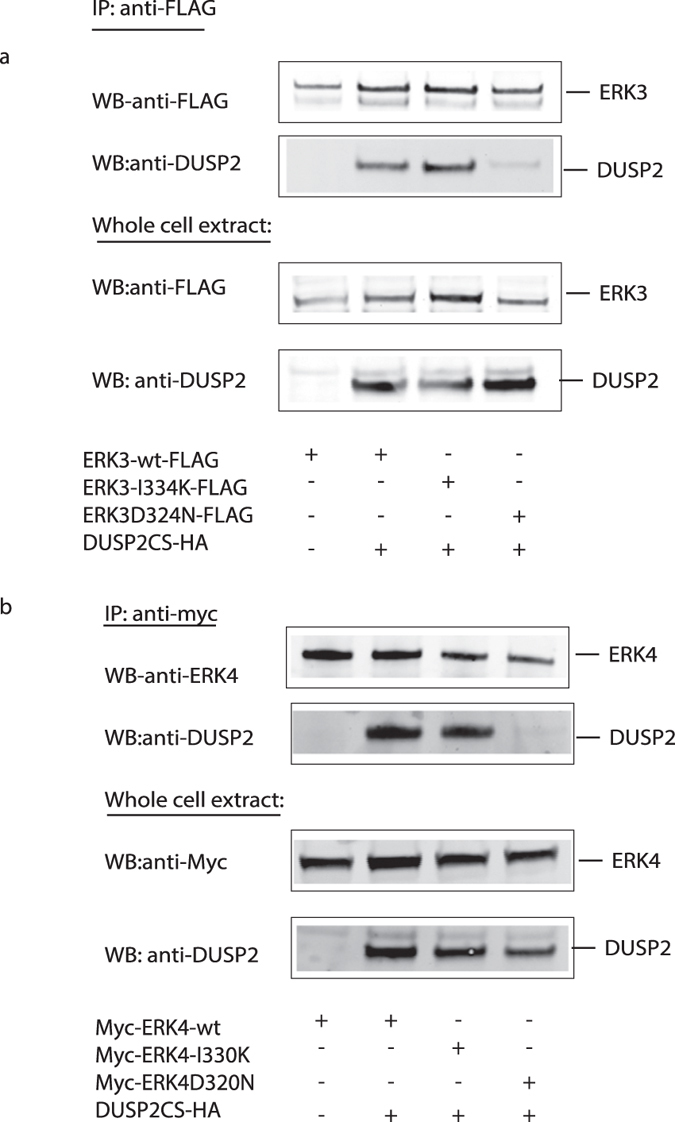
The ability of ERK3 and ERK4 to interact with DUSP2 is dependent on the common docking (CD) motif, but does not require an intact FRIEDE motif. (**a**) NCI-H1299 cells were co-transfected with expression vectors encoding either FLAG-tagged wild-type ERK3 (ERK3wt), a common-docking site ERK3 mutant (ERK3D324N), or a FRIEDE ERK3 mutant (ERK3I334K) together with an expression vector encoding an myc-tagged catalytically inactive mutant of DUSP2 (DUSP2CS). After 24 h, cells were lysed and FLAG-tagged ERK3 proteins were immunoprecipitated using an anti-FLAG antibody. Any co-immunoprecipitated DUSP2 was detected by Western-blotting using a DUSP2 specific antibody (upper panel). The expression levels of ERK3 and DUSP2 proteins in the cell lysates were analysed by Western-blotting using an anti-FLAG or anti-DUSP2 specific antibody respectively (lower panels). (**b**) NCI-H1299 cells were co-transfected with expression vectors encoding either myc-tagged wild-type ERK4 (ERK4wt), a common-docking site ERK4 mutant (ERK4D320N), or an ERK4 mutant in which the FRIEDE motif was rendered non-functional FRIEDE (ERK4I330K) together with an expression vector encoding HA-tagged catalytically inactive DUSP2 (DUSP2CS). After 24 h, cells were lysed and myc-tagged ERK3 proteins were immunoprecipitated using an anti-myc antibody. Any co-immunoprecipitated DUSP2 was detected by Western-blotting using a DUSP2 specific antibody (upper panel). The expression levels of ERK4 and DUSP2 proteins in the cell lysates were analysed by Western-blotting using an anti-myc or anti-DUSP2 specific antibody respectively (lower panels). Unprocessed original scans of the blots are shown in Supplementary Fig. 1

We recently discovered a novel sequence motif located within the L16 extension lying C-terminal to the CD domain in both ERK3 and ERK4. This “FRIEDE” motif is absolutely required for both ERK3 and ERK4 to bind to MK5/PRAK and a single isoleucine to lysine substitution (I334K in ERK3 and I330K in ERK4) totally abrogates binding, activation, and nuclear to cytoplasmic translocation of MK5 by both kinases^8^. To determine if this motif plays any role in the binding between either ERK3 or ERK4 and DUSP2 we co-expressed Myc-tagged ERK3 or ERK4, either wild-type, with the FRIEDE motif mutation or a mutation in the CD domain, together with inactive DUSP2 (DUSP2CS). Clearly both the wild-type and the FRIEDE mutant forms of ERK3 and ERK4 bind to DUSP2, while mutation of the CD domain completely abrogated the binding (Fig. 5a and b). We can conclude that the FRIEDE motifs and the CD domains function completely autonomously in the binding of both ERK3 and ERK4 to either their specific substrate MK5, or the regulatory protein phosphatase DUSP2 respectively.

### DUSP2 is relocalized from the nucleus to the cytoplasm on binding to ERK3 or ERK4

**Figure 6:**
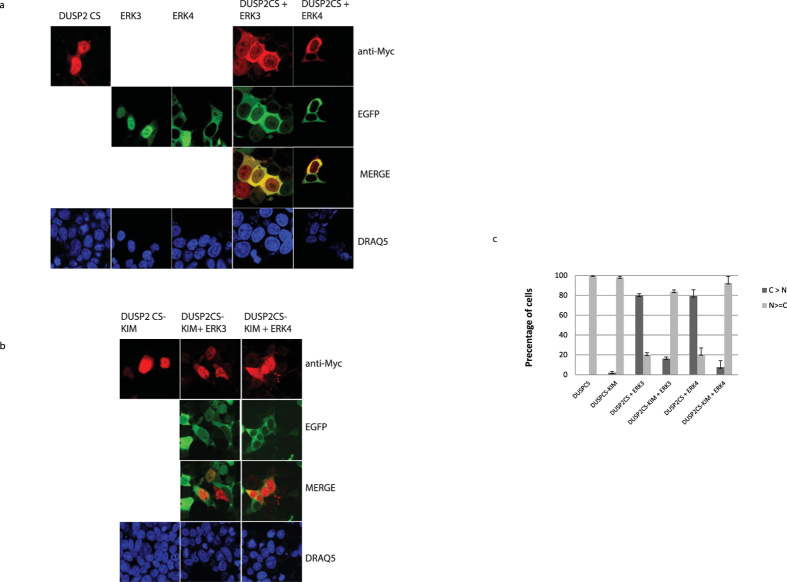
Coexpression of ERK3 or ERK4 results in cytoplasmatic relocalisation of DUSP2. (**a**) HEK293 cells were transfected with expression plasmids encoding myc-tagged catalytically inactive DUSP2 mutant (DUSP2CS), EGFP-tagged ERK3, ERK4 or a combination of DUSP2CS and ERK3 or DUSP2CS and ERK4. After 24 h the cells were fixed, EGFP fluorescence was visualized directly, and myc-tagged DUSP2 was visualized by staining with an anti-myc antibody (9E10) and Alexa-594 anti mouse antibody. (**b**). HEK293 cells were cotransfected with expression plasmid encoding either myc-tagged kinase interaction motif-deficient mutant of inactive DUSP2 (DUSP2CS-KIM) and EGFP-tagged ERK3 or myc-tagged kinase interaction motif-deficient mutant of inactive DUSP2(DUSP2CS-KIM) and EGFP-tagged ERK4. The cell was fixed and protein visualized as in A. (**c**) HEK 293 cells were co-transfected with the indicated expression vectors, and EGFP-ERK3, EGFP-ERK4, myc-DUSP2CS and myc-DUSP2CS-KIM were visualized as above. In all, more than 100 cells expressing DUSP2CS or DUSP2CS-KIM alone or together with either EGFP-ERK3 or EGFP-ERK4 from three independent transfections were counted, and the distribution of DUSP2 protein was scored in percentages. The results are presented as the percentages of cells in which myc-DUSP2 was predominantly cytosolic (C > N), and the mean values with the associated SD are shown. In all experiments, 10 different fields of cells were examined, and the representative images are as shown in Fig.6a and b.

Several studies have shown that dual-specificity phosphatases are able to influence the subcellular distribution of MAP kinases^16^,^21^. We have previously shown that ERK3 predominantly localizes to the cell nucleus whereas ERK4 localizes to the cytoplasm. However, both kinases are able to shuttle between the nucleus and the cytoplasm. To investigate if DUSP2 could affect the subcellular distribution of ERK3 and ERK4, we overexpressed EGFP-fusion proteins of either ERK3 or ERK4 alone or together with a catalytically inactive version of DUSP2. In agreement with a previous report^13^, ectopically expressed DUSP2CS is localized to the cell nucleus (Fig. 6a). Interestingly, upon co-expression of both ERK3 and ERK4, the phosphatase relocalises from the nucleus to the cytoplasm, and co-expression of ERK3 and DUSP2 led to a relocalization of both proteins from the nucleus to the cytoplasm. This is reminiscent of the effects of co-expressing ERK3 and ERK4 with its binding partner and substrate MK5, which also causes both proteins to relocalise to the cytoplasm^22^,^23^ In contrast, the localization of a phosphatase-inactive DUSP2 KIM mutant remained predominantly nuclear upon co-expression with either ERK3 or ERK4 (Fig. 6b and c). We conclude that KIM-dependent protein-protein interactions between DUSP2 and either ERK3 or ERK4 cause the redistribution of DUSP2 from nucleus to cytoplasm.

### DUSP2 is an unstable protein and its half-life is increased on binding to ERK4

**Figure 7:**
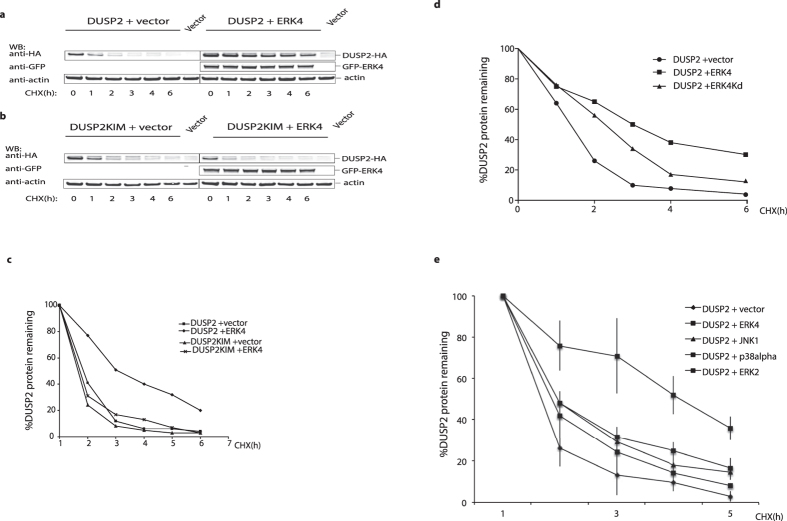
ERK4 stabilizes the DUSP2 protein *in vivo*. (**a,b**) HeLa cells were co-transfected with expression vectors encoding either DUSP2-HA (**a**) or DUSP2KIM-HA (**b**) together with either an empty expression vector or a plasmid encoding GFP-ERK4. After 24 h, cells were treated with 10 μM cycloheximide (CHX) for the indicated time periods before cell harvesting and lysis. The levels of DUSP2-HA or DUSP2KIM-HA in whole cell extracts were analysed by Western blotting using an anti-HA antibody. Levels of GFP-ERK4 and endogenous actin in the cell extracts were analysed by Western blotting using antibodies against GFP and actin, respectively. Unprocessed original scans of the blots are shown in Supplementary Fig. 1 (**c**) The intensities of the signals corresponding to the DUSP2 and DUSP2KIM bands in both a and b, were then quantified using the Odyssey infrared imaging System. The band intensities at the indicated time points are then expressed graphically as a percentage of the intensity at time zero. (**d**) A kinase-dead mutant of ERK4 stabilizes DUSP2 less efficiently than wild-type ERK4. Experiments were performed as described in (a–c). **(e**) Co-expression of classical MAP kinases does not lead to stabilization of the DUSP2 protein. HeLa cells were co-transfected with an expression vector encoding HA-tagged wild type DUSP2 together with expression vectors encoding either myc- tagged ERK4, ERK2, p38α or JNK1. Cycloheximide time course experiments, followed by Western blot analyses and quantification were performed exactly as described in a-c. The average of three independent experiments is shown including standard deviation. The experiments (**a–d**) were performed three times with identical results and the results of a single representative experiment are shown.

Many MKPs are relatively unstable proteins and their half-life can be modified by either site-specific phosphorylation or binding to their cognate substrates. For instance, DUSP1/MKP-1 is stabilized by ERK2-dependent phosphorylation of C-terminal residues, while both DUSP5 and DUSP6/MKP-3 are stabilized on binding to ERK2^24^,^25^,^26^. To explore the relationship between DUSP2 stability and ERK4 we expressed DUSP2 either in the presence or absence of wild-type ERK4 and monitored the loss of the DUSP2 protein on addition of the protein synthesis inhibitor cycloheximide. DUSP2 levels declined rapidly following cycloheximide addition, indicating that the protein is highly unstable *in vivo*. However, co-expression of ERK4 significantly increased the half-life of DUSP2 (Fig. 7a). This was strictly dependent of the integrity of the KIM motif within DUSP2, as a KIM mutant was not stabilized on expression of ERK4 (Fig. 7b). Interestingly, a kinase dead mutant of ERK4 was not as efficient as wild-type ERK4 in stabilizing DUSP2 despite being expressed at similar levels, indicating that catalytic activity and possibly the phosphorylation of DUSP2 by ERK4 might play some role in increasing DUSP2 stability (Fig. 7d). Finally, only co-expression of wild-type ERK4 had a significant effect on DUSP2 stability as expression of either ERK2, p38α or JNK1 caused only a minor increase in the half-life of DUSP2 when compared with ERK4 (Fig. 7e). Co-expression of ERK3 did not give rise to similar increase in DUSP2 half-life (data not shown).

### Expression of wild-type DUSP2 reduces the phosphorylation of the activation loop within MK5 by both ERK3 and ERK4

**Figure 8:**
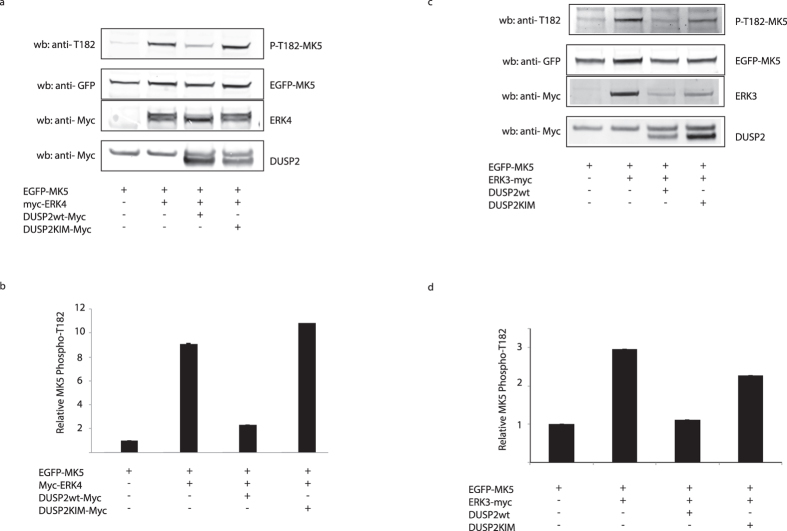
Expression of DUSP2 inhibits the ERK3 and ERK4-mediated phosphorylation of MK5. NCI-H1299 cells were co-transfected with expression plasmids encoding either myc-tagged ERK4 (**a** and **b**) or myc-tagged ERK3 (**C** and **D**) together with an expression vector encoding an EGFP-MK5 fusion protein and either an empty expression vector or plasmids encoding either wild-type DUSP2 or a KIM mutant of DUSP2 as indicated. After 24 h, the cells were lysed and MK5 phosphorylated at Thr-182 was detected by Western-blotting using a phospho-specific anti-Thr182 MK5 antibody (**a** and **c**). The expression of MK5, ERK3, ERK4 and either wild-type DUSP2 or the DUSP2 KIM mutant were verified by Western blotting of the cell lysates using appropriate antibodies (bottom panels in **a** and **c**). Unprocessed original scans of the blots are shown in Supplementary Fig. 1. The data in a and c and two additional replicate experiments were then quantified using the Odyssey infrared imaging System. The relative intensity of the p-Thr182 MK5 bands are calculated using the band intensity from cells transfected with EGFP-MK5 alone as the reference and mean values are presented with associated errors (**b** and **d**).

The best characterized protein substrate for the atypical MAPKs ERK3 and ERK4 is MK5 (PRAK)^22^,^23^,^27^,^28^. The activation of MK5 by ERK3 and ERK4 is mediated by phosphorylation of T182 within the activation loop of MK5 and this is dependent on specific interactions between ERK3/ERK4 and MK5 mediated by the FRIEDE motif within the C-terminal domain of these atypical MAPKs^8^. We have previously shown that phosphorylation of S186 within ERK4 is absolutely necessary for its ability to interact with, phosphorylate, and activate MK5^6^. To determine the effects of DUSP2 on MK5 activation loop phosphorylation, an EGFP-MK5 fusion protein was co-expressed with myc-tagged wild-type ERK4 in the absence or presence of either wild-type DUSP2 or the KIM mutant of DUSP2. The cell lysates were analysed by Western blotting using a specific antibody that specifically recognises MK5 when phosphorylated on T182. As previously demonstrated, MK5 was phosphorylated on T182 upon co-expression of ERK4. However, coexpression of wild-type DUSP2, but not the KIM mutant of DUSP2 significantly reduced the levels of T182 phosphorylation (Fig. 8a and b). This strongly indicates that the ability of DUSP2 to dephosphorylate S186 within ERK4 results in disruption of its interaction with and subsequent activation of MK5 (Fig. 8a and b). Identical results were obtained when EGFP-MK5 was co-expressed with wild-type ERK3 and either wild-type or the KIM mutant of DUSP2 (Fig. 8c and d). Overall these results clearly indicate that DUSP2 is capable of negatively regulating signal transduction through the ERK3/4-MK5 pathway in mammalian cells.

## Discussion

The atypical MAP kinases ERK3 and ERK4 are unusual in that the signature activation motif T-X-Y, which is conserved in all classical MAPKs, is replaced by S-E-G in which the serine residue is the sole phospho-acceptor. Despite this difference, it is now clear that phosphorylation of S189 and S186 in ERK3 and ERK4, respectively, is both essential and sufficient for the ability of these kinases to interact with, phosphorylate, and activate their best characterised downstream substrate the MK5 (PRAK) protein kinase both *in vitro* and *in vivo*^6^,^29^. The fact that ERK3 and ERK4 require phosphorylation of a single serine residue dispenses with the requirement that the upstream activating kinase be a member of the MEK (or MKK) family of dual–specificity Thr/Tyr protein kinases. Indeed, where tested these enzymes have negligible activity towards the S-E-G motif in ERK3 *in vitro*^30^, suggesting that this signalling pathway may employ a non-canonical activation mechanism. This idea is supported by the recent finding that members of the class I p21-activated kinases (PAks) mediate robust phosphorylation of the S-E-G motif resulting in enzymatic activation of both ERK3 and ERK4 and downstream activation of MK5/PRAK *in vivo*^4^,^5^. Despite the emergence of this novel Pak1-ERK3/ERK4-MK5 signalling axis, many questions remain to be answered about the regulation of this atypical MAPK pathway. In particular, studies of the kinetics of ERK3/ERK4 phosphorylation have shown no particular correlation with any physiological agonists or stress conditions normally associated with MAPK pathway activation and the phosphorylation of S189 in ERK3 can persist in quiescent cells in the absence of growth factor stimulation^29^.

One important regulatory mechanism employed to control the activity of MAPKs is the regulated and specific dephosphorylation of the T-X-Y motif by a family of dual-specificity MAPK phosphatases^9^. Despite the non-canonical activation mechanism employed by these kinases the “common docking” (CD) domain that mediates protein-protein interactions between classical MAPKs and a range of downstream substrates and regulators is conserved in both ERK3 and ERK4^8^. However, the interaction between both ERK3 and ERK4 and their substrate MK5 is mediated by the FRIEDE motif, a distinct and novel MAPK interaction site within the C-terminal domain of both kinases^8^. Given the importance of the CD domain in the interactions between classical MAPKs and regulatory phosphatases^7^ we screened the ten members of the dual-specificity MKP family for their ability to interact with ERK3 and ERK4 using the yeast 2-hybrid assay. This revealed that a single member of this family, DUSP2, was able to bind to ERK3 and ERK4 and that this interaction was absolutely dependent on the integrity of both the CD domain and the conserved kinase interaction motif (KIM) located within the amino terminal non-catalytic domain of DUSP2.

DUSP2 was originally characterised as a mitogen-inducible nuclear protein tyrosine phosphatase in T cells^13^ and subsequently shown to have catalytic activity towards both the classical ERK1/2 MAPKs and to a lesser extent the p38 stress-activated MAPKs^11^,^12^. Our results indicate that DUSP2 binds as strongly, if not more strongly, to both ERK3 and ERK4 compared to ERK2 *in vitro. Moreover* our experiments show that this interaction is direct and results in the efficient dephosphorylation of the serine phospho-acceptor residue within the activation loop of ERK3 and ERK4. Although binding to ERK2 results in a significant increase in the catalytic activity of DUSP2 *in vitro,* no such increase in activity is seen on incubation with either ERK3 or ERK4. However, although catalytic activation is a feature of several MKPs it is not a universal property of these proteins and several phosphatases including DUSP5 and DUSP10 are not activated on binding to their *bona fide* MAPK substrates^9^.

Several MKPs are relatively unstable proteins *in vivo* and their turnover is mediated by proteosomal degradation. In certain cases, such as DUSP1/MKP-1 and DUSP4/MKP-2 the interaction of the phosphatase with MAPK substrate results in phosphorylation of the former and a decrease in the rate of protein turnover. Interestingly, we found that the co-expression of ERK4 and DUSP2 increased the stability of the latter and that this stabilization was dependent on both the KIM motif in DUSP2 and on the catalytic activity of ERK4, a possible indication that the phosphorylation of DUSP2 might be involved in stabilisation of the protein. In support of the latter notion we have shown that wild-type ERK4 can phosphorylate DUSP2 *in vitro* and future studies will be aimed at identifying the relevant site(s) of modification and determining their influence on DUSP2 stability. Finally, we have clearly demonstrated that the expression of DUSP2 is capable of supressing the ERK3 or ERK4-dependent phosphorylation of MK5, indicating that DUSP2 may regulate the Pak-ERK3/ERK4-MK5 signalling axis.

Although DUSP2 can suppress ERK3 and ERK4 mediated activation of ectopically expressed MK5, we have not been able to detect any influence of DUSP2 expression on endogenous MK5 activity (data not shown). These experiments were performed using siRNA knockdown of DUSP2 in activated Jurkat T-cells were DUSP2 is readily induced in response to activation of the T-cell receptor and the endpoint assay was a change in MK5 activation loop phosphorylation assayed in while cell extracts by Western blotting. Our failure to see any changes in MK5 phosphorylation could be because DUSP2 is acting on a subpopulation of ERK3 perhaps in a discrete subcellular compartment. Furthermore, we and others have shown that co-expression of MK5 increases the phosphorylation of S189 and S186 in ERK3 and ERK4 respectively^6^,^29^, suggesting that perhaps the activation-loop of ERK3 and ERK4 is not accessible for dephosphorylation when complexed with MK5. Thus, DUSP2 may be important for the suppression of ERK3 (or ERK4) activity in respect of other as yet unidentified targets or may modulate kinase activity in certain subcellular compartments. Finally, there may be cell types where DUSP2 plays a more prominent role in the regulation of MK5 activation. However, so far we have been unable to identify other cell lines in which we can detect endogenous co-expression of ERK3/ERK4, DUSP2 and MK5.

In conclusion, our results strongly suggest that the atypical MAPKs ERK3 and ERK4 are *bona fide* substrates of DUSP2 and this represents a significant step forward in our understanding of these atypical MAPK signalling pathways. In addition, the discovery that DUSP2 can regulate ERK3 and ERK4 provides an additional tool with which to modulate the activities of these pathways *in vivo*. Finally, despite it being amongst the first of the dual-specificity MKPs to be identified in mammalian cells, the physiological function(s) of DUSP2 are rather poorly understood^31^. Mice lacking DUSP2 present a phenotype of reduced inflammatory responses in the ‘K/BxN’ model of rheumatoid arthritis^32^. However, these effects are difficult to reconcile with observed changes in the activities of the classical MAPK pathways when analysed in DUSP2 knockout cells and tissues. Thus far the possible role(s) of ERK3 and ERK4 signalling in the immune system and inflammation have not been widely explored although recent work suggests that ERK3 plays some role in thymocyte survival and positive selection^33^,^34^,^35^. The discovery that ERK3 and ERK4 may be regulatory targets for DUSP2 *in vivo* should prompt future studies that may link ERK3 and ERK4 with these inflammatory endpoints and lead to a fuller understanding of the DUSP2 knockout phenotype.

## Methods

### Reagents

All chemicals were purchased from SigmaAldrich.

### Antibodies

The polyclonal antibody against DUSP2 was raised in sheep using recombinant GST-DUSP2 as an antigen. The polyclonal antibodies against ERK4 and MK5 phosphorylated at threonine 182 have been described previously^6^,^23^ and were kindly provided by Philip Cohen (MRC protein phosphorylation Unit, Dundee, UK). The polyclonal GFP antibody (sc-8334) and a polyclonal GST (Z-5) antibody (sc-459) were purchased from Santa-Cruz Biotechnology (Santa Cruz, CA). The monoclonal ERK3 antibody (clone 4C11) was purchased from Abnova (Taiwan). Anti-FLAG (M2-conjugated) was purchased from Sigma-Aldrich. The polyclonal antibody recognizing ERK3 phosphorylated at Serine 189 (AP3098a) was purchased from Abgent (San Diego, Ca, USA). Monoclonal anti-myc antibody (9E10) was from Cancer Research-UK. The rabbit polyclonal anti-EGFP antibody used for immunoprecipitation of EGFP fusion proteins was kindly provided by T. Johansen (University of Tromsoe). Alexa Fluor^®^ 680 goat anti-rabbit IgG (A21076), Alexa Fluor® 680 goat anti-mouse IgG (A-21057) and Alexa Fluor^®^ 680 donkey anti-sheep IgG (A-21102) were purchased from Life technologies. IRDye 800 conjugated anti-HA (glutathione transferase) (600-132-200) and anti-Myc antibodies (600-132-215), IRDye 800CW conjugated affinity-purified anti-mouse IgG (610-131-121) and IRDye 800CW conjugated affinity-purified anti-rabbit IgG (611-131-122) were purchased from Rockland. Monoclonal Anti-CD3 antibody (#555329) was purchased from BD Bioscience.

### Cell lines and transfection

HeLa cells (ATCC CCL2) were maintained in Eagle’s minimum essential medium supplemented with 1x nonessential amino acids (Invitrogen), 10% fetal bovine serum (FBS), 2 mM L-glutamine, penicillin (100 units/ml), and streptomycin (100 μg/ml). HEK 293 cells (ATCC CRL-11268) were maintained in Dulbecco’s modified Eagle’s medium supplemented with 10% FBS, 2 mM L-glutamine, penicillin (100 U/ml), and streptomycin (100 μg/ml). NCI-H1299 cells (ATCC CRL 5803) were maintained in RPMI 1640 medium supplemented with 10% FBS, 2 mM L-glutamine, penicillin (100 units/ml), and streptomycin (100 μg/ml). Jurkat E6.1 cells (ATCC TIB152) were maintained in RPMI 1640 containing 10% FBS. Lipofectamine Plus (Invitrogen) reagent was used to transfect the cells according to the manufacturer’s instructions. Jurkat cells were transfected using Amaxa nucleofection (Lonza Group Ltd) according to the manufacturer’s protocol.

### DNA constructs

To generate pGEX-mDUSP2, full-length mDUSP2 was amplified by PCR from an EST-clone and inserted into the NdeI/XhoI sites of pGEX-5X-3 (GE-Healthcare) using the following PCR primers 5′-gaggaattcatatggggctggagacggcgtgcgag-3′ and 5′-ccgctcgagtcagtgacacagcacctgggtctg-3′. pGEX-mDUSP2CS was generated by site-directed mutagenesis using the Quickchange method (Stratagene) and the primer (only forward shown) 5′-gggccgggtgctggtgcacagccaagctggcatctctcgc-3′. To make a kinase interaction motif (KIM)-deficient version of pGEX-mDUSP2 (pGEX-mDUSP2KIM), a construct encoding a protein where three subsequent arginine residues at position 60, 61 and 62 were replaced by alanine, serine and alanine, respectively, was generated by site-directed mutagenesis using primer 5′-ccctggaacgcgctgctggcgtcggctgcgcgcggcacccccgcc-3′ (forward). For expression epitope-tagged DUSP2 in mammalian cells human DUSP2 was amplified using the following primers; 5′-gaggaattcatatggggctggaggcggcgcgcgag -3′ and 5′-ccgctcgaggtgacacagcacctgggtctc-3′ and subcloned as a EcoRI/XhoI fragment into a modified pSG5 vector encoding either a single Myc-tag or HA-tag at the C-terminus of the expressed protein. The expression constructs for EGFP-MK5, epitope-tagged p38α and JNK-1, epitope-tagged wild-type and mutant forms of ERK2, ERK3, and ERK4 have been described earlier^8^,^14^,^22^,^23^,^36^

### Yeast two-hybrid assay

Expression plasmids encoding GAL4 DB (DNA-binding domain) and AD (activation domain) fusion proteins were transformed into the *Saccharomyces cerevisiae* strains PJ69-2A(MATa) and Y187(MATα) respectively, using the Frozen-EZ Yeast Transformation II (Zymo Research), and plated out on to suitable drop-out medium, according to the manufacturer′s protocol. The yeast-mating procedure and semi-quantitative β-galactosidase assay were performed as described previously^14^.

### Recombinant ERK3 and ERK4

The expression and purification of recombinant ERK3 and ERK4 from baculovirus-infected Sf-9 insect cells was performed exactly as described previously^6^,^22^.

### Expression of GST fusion proteins in E.coli

GST fusion proteins of wild-type and mutants of murine DUSP2 were expressed in E.coli (BL21) by induction with 0.1 mM isopropyl-1-thio-β-D-galactopyranoside at 18 °C for 20 h (overnight) and purified using glutathione-sepharose using standard techniques. SDS PAGE and Coomassie Blue staining were used to analyze both the expression and yield of the fusion proteins.

### GST pull down assays

2 μg recombinant ERK4 and ERK3 were precleared prior to the pull-down using 2 μg GST-protein and 30 μl Glutathione-Sepharose (Amersham Biosciences AB) (50% slurry equilibrated in a buffer containing 50 mM Tris/HCl pH 7.5, 1 mM EGTA, 1 mM EDTA, 1% (w/v) Triton-X 100, 1 mM sodium orthovanadate, 50 mM sodium fluoride, 5 mM sodium pyrophosphate and 0.27 M sucrose) for 1 h at 4 °C. Direct interaction between DUSP2 and the atypical MAP kinases was detected by incubating 2 μg of recombinant ERK4 or ERK3 with 2 μg purified GST-DUSP2, GST-DUSP2KIM, GST-DUSP2CS or GST in 200 μl of the above buffer for 2 h at 4 °C. After addition of 30 μl Glutathione-Sepharose (50% slurry equilibrated in the above buffer) the samples were incubated for an additional hour. The beads were washed 5 times with the above buffer and once in 50 mM Tris PH 7.5 and then resuspended in 40 μl 2 x SDS-sample buffer. GST-DUSP2 fusions and co-precipitated ERK4 and ERK3 were detected by SDS PAGE and Western blotting using GST antibody and ERK4 or ERK3 antibodies respectively.

### Coimmunoprecipitation

Immuno precipitation and co-immuno precipitation of both endogenous and epitope-tagged proteins were performed as previously described^23^.

### Immunoblotting

For detection of endogenous ERK3, ERK4 and epitope-tagged DUSP2, ERK3, ERK4, and MK5 in transfected cells and co-immunoprecipitation experiments, the samples were analyzed by SDS-PAGE (4–12% NUPAGE, Life Technologies), transferred to a nitrocellulose membrane (Amersham Biosciences), and probed with either anti-ERK3 (1:500), anti-ERK4 (1:1000), anti-Myc (1:200), anti-HA (1:1000), anti-ERK4 (1:1000), anti-P-T182-MK5 (1:500), anti-EGFP (1:200), anti-S186-ERK4 (1:500), anti-phospho-S189-ERK3 (1:500) anti-DUSP2 (1:2000) or anti-GFP IRDye^TM^ 800-conjugated (1:5000) antibodies. Detection and quantification were performed either directly using anti-GFP IRDye^TM^ 800-conjugated antibody or by conjugated goat anti-mouse IgG (H&L) or IRDye 800CW-conjugated goat anti-rabbit IgG (H&L) (1:5000) or Alexa Fluor 680-conjugated donkey anti-sheep IgG (H&L) (1:5000) Protein bands were visualized using a Li-Cor Odyssey infrared imager and Immunoblots are representative of three independent experiments. Protein molecular mass was estimated using the MagicMark Western protein standard (Life technologies). Unprocessed original scans of blots are shown in Supplementary Fig. 1.

### Phosphatase Assays

The phosphatase activity of recombinant murine DUSP2 in the absence or presence of recombinant ERK2, ERK3, or ERK4 was determined by measuring the conversion of *para*-Nitrophenyl Phosphate (*p*-NPP) to *para*-Nitrophenol in a spectrophotometric assay. 10 μg of GST-DUSP2, GST-DUSP2KIM, GST-DUSP2CS or GST were transferred to 96 Well microtiter plates with or without 10 μg ERK2, ERK3, or ERK4. 20 mM *p-*NPP in 200 μl buffer containing 50 mM imidazole and 10 mM dithiothreitol was added per well and the absorbance at 405 nm was measured immediately. The plates were then incubated at 23 °C in the dark for 3 h, and the absorbance at 405 nm was measured every 20 minutes.

### Cell Staining and Microscopy

Determination of the subcellular localization of GFP fusion proteins, Myc-tagged DUSP2 and the visualization of cell nuclei was performed as described previously^22^. Images were collected using a Zeiss LSM510 confocal laser-scanning microscope and processed using Adobe Photoshop. For cell counting experiments several fields of cells from each transfection or co-transfection were examined and at least one hundred cells were scored for the subcellular localization of the protein(s) of interest.

## Additional Information

**How to cite this article:** Perander, M. *et al*. Regulation of atypical MAP kinases ERK3 and ERK4 by the phosphatase DUSP2. *Sci. Rep.* **7**, 43471; doi: 10.1038/srep43471 (2017).

**Publisher's note:** Springer Nature remains neutral with regard to jurisdictional claims in published maps and institutional affiliations.

## Supplementary Material

Supplementary Information
