# Supplementary material for: Regulation of atypical MAP kinases ERK3 and ERK4 by the phosphatase DUSP2
Source: Sci Rep. 2017 Mar 2;7:43471. doi: 10.1038/srep43471 (PMC5333157; doi:10.1038/srep43471)

Regulation of atypical MAP kinases ERK3 and ERK4 by the phosphatase DUSP2

[2]Maria Perander, [1]Rania Al-Mahdi, [1]Thomas C. Jensen [1]Jennifer A.L. Nunn, [1]Hanne Kildalsen, [1]Bjarne Johansen, [3,4]Mads Gabrielsen, [3*]Stephen M. Keyse and [1*]Ole-Morten Seternes.

[1]Department of Pharmacy, [2]Department of Medical Biology, UiT The Arctic University of Norway, N-9037 Tromsø, Norway. and [3]Cancer Research UK Stress Response Laboratory, Medical Research Institute, Division of Cancer Research, Jacqui Wood Cancer Centre, James Arrot Drive, Ninewells Hospital and Medical School, Dundee DD1 9SY, United Kingdom, [4]present address; Beatson Institute for Cancer Research, Garscube Estate, Switchback Road Glasgow G61 1BD, United Kingdom.

[*] To whom correspondence should be addressed: Ole-Morten-Seternes@uit.no or s.m.keyse@dundee.ac.uk

Supplementary Figure Legends

Supplementary Figure 1: Unprocessed Western blot scans.

Figure 2A and B

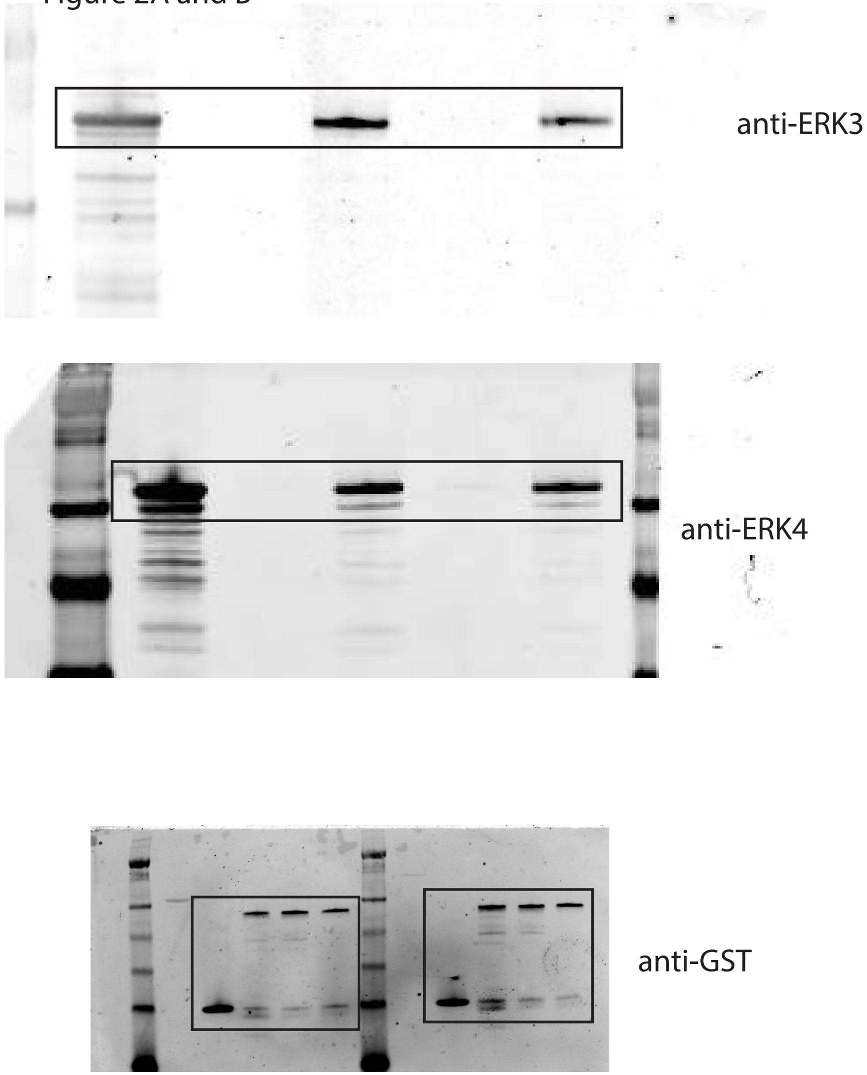

Figure 2C

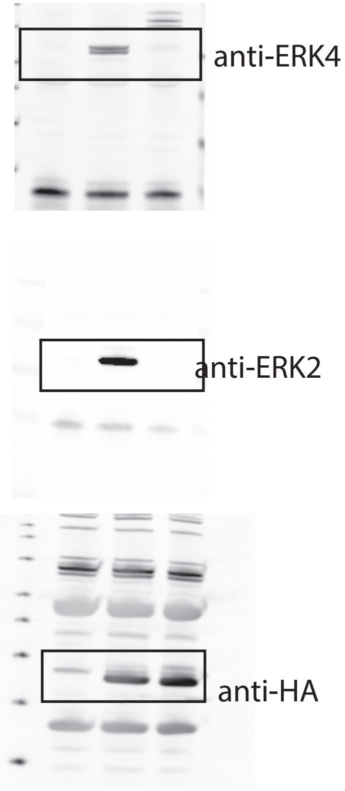

Figure 2D

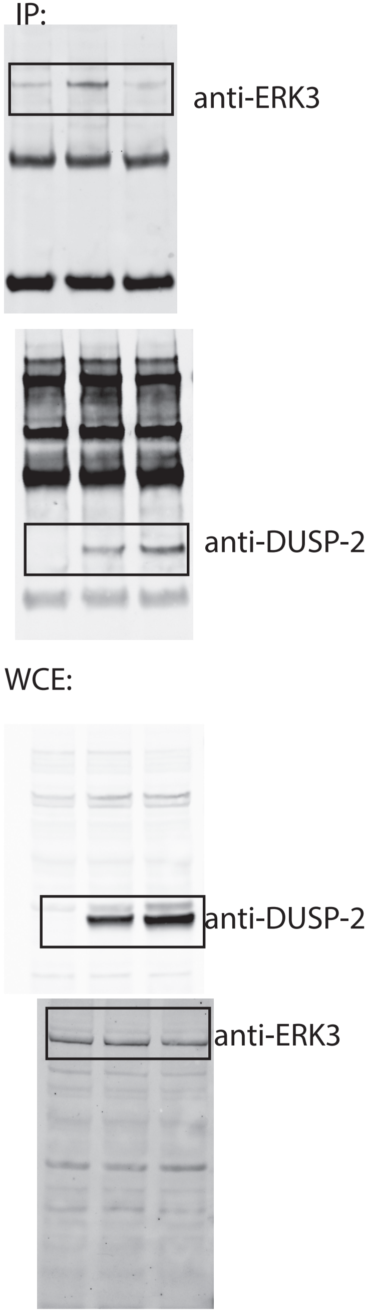

Figure 2E

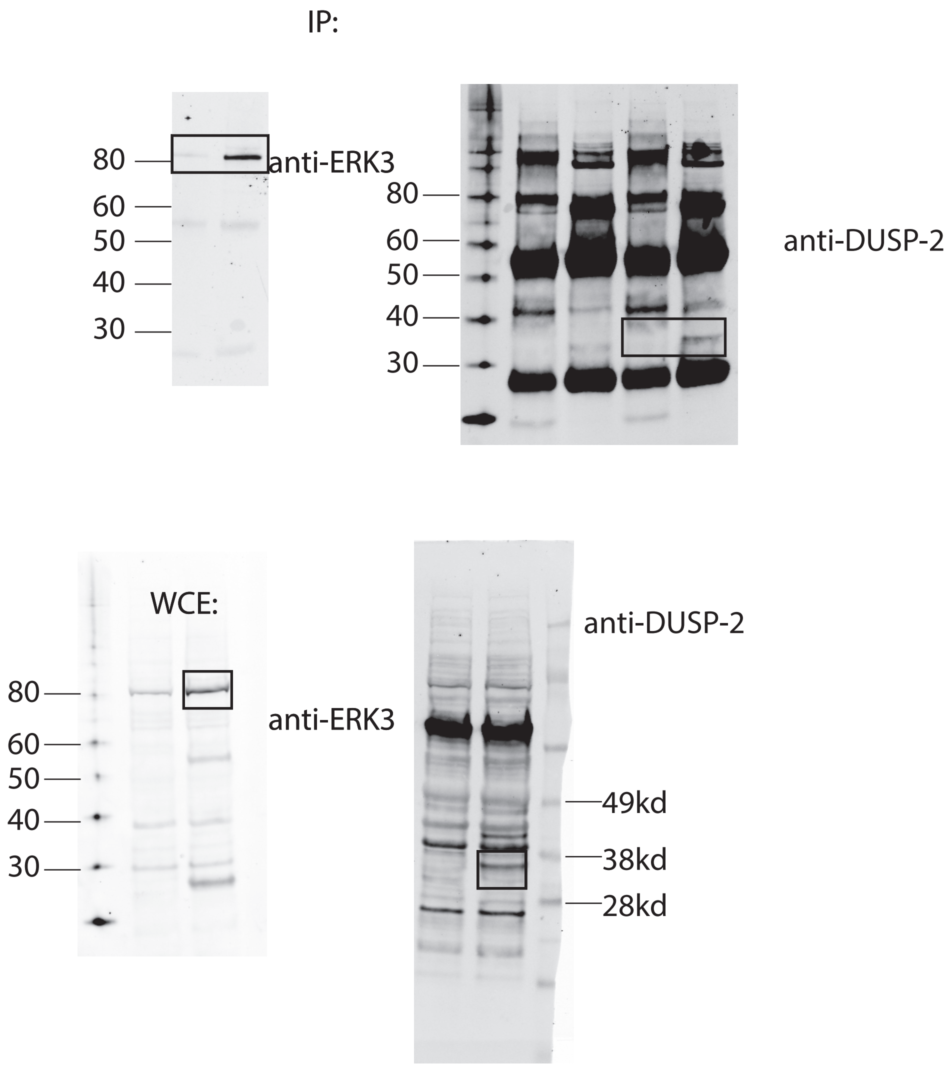
IP:
80
60
50
40
30
anti-ERK3
80
60
50
40
30
anti-DUSP-2
WCE:
80
60
50
40
30
anti-ERK3
anti-DUSP-2
49kd
38kd
28kd

Figure 4 A

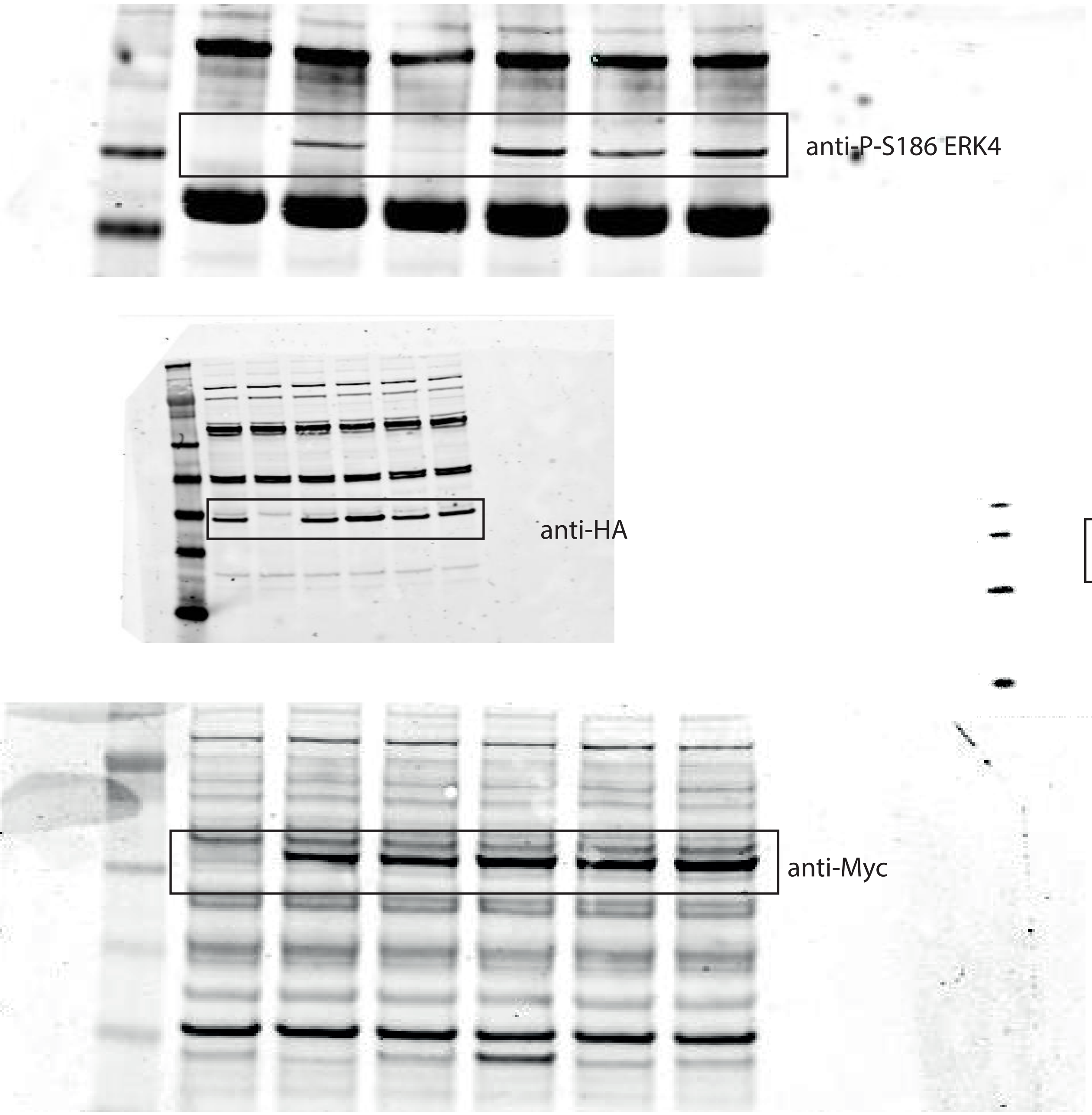

Figure 4 B

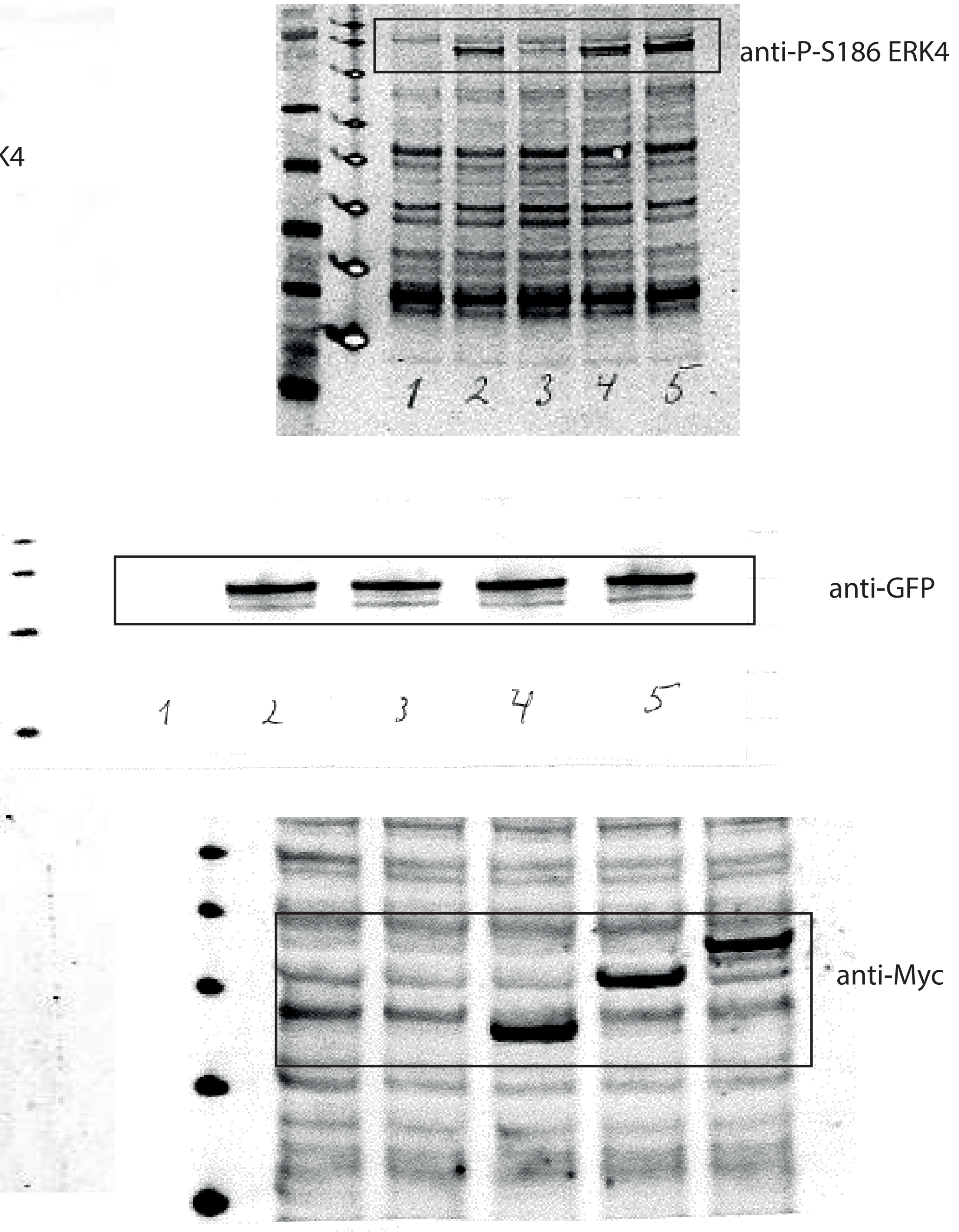

FIGURE 4C

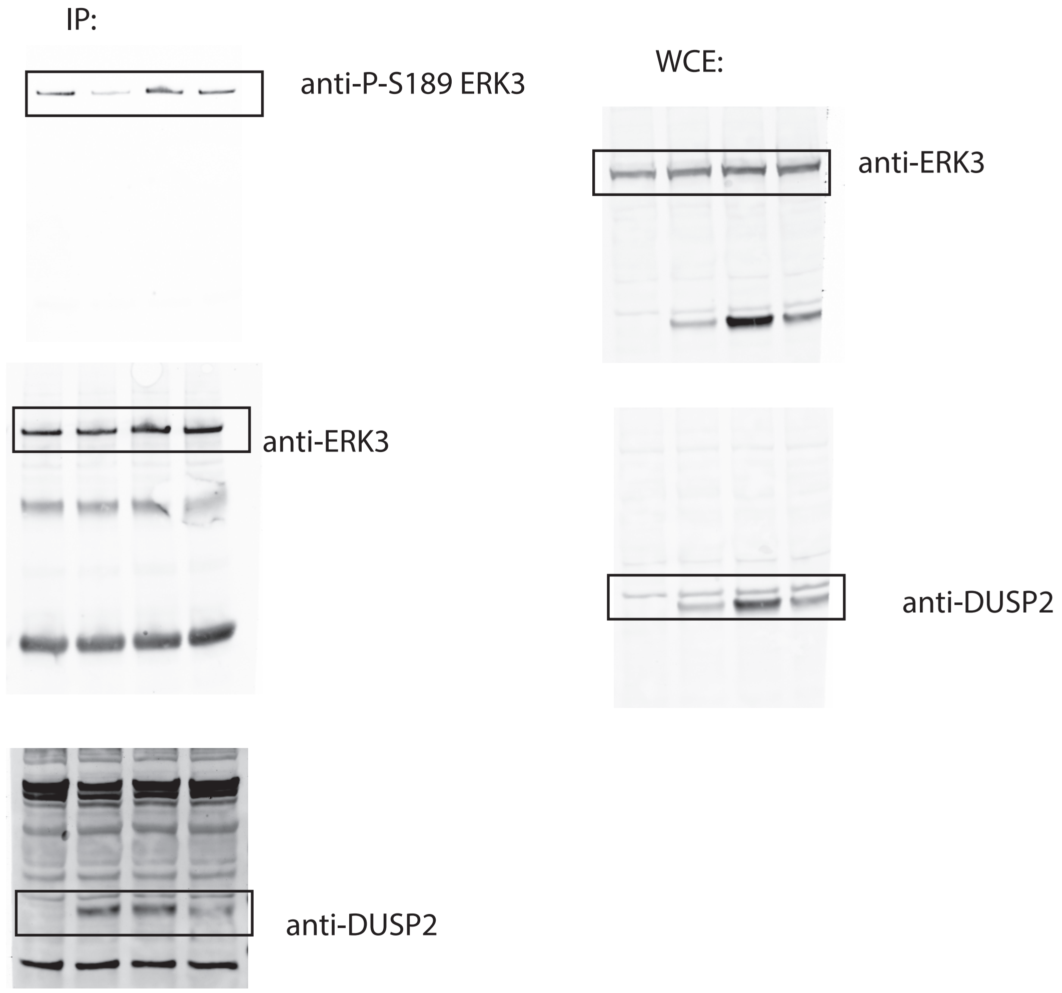

IP:
anti-P-S189 ERK3
anti-ERK3
anti-DUSP2
WCE:
anti-ERK3
anti-DUSP2

FIGURE 5

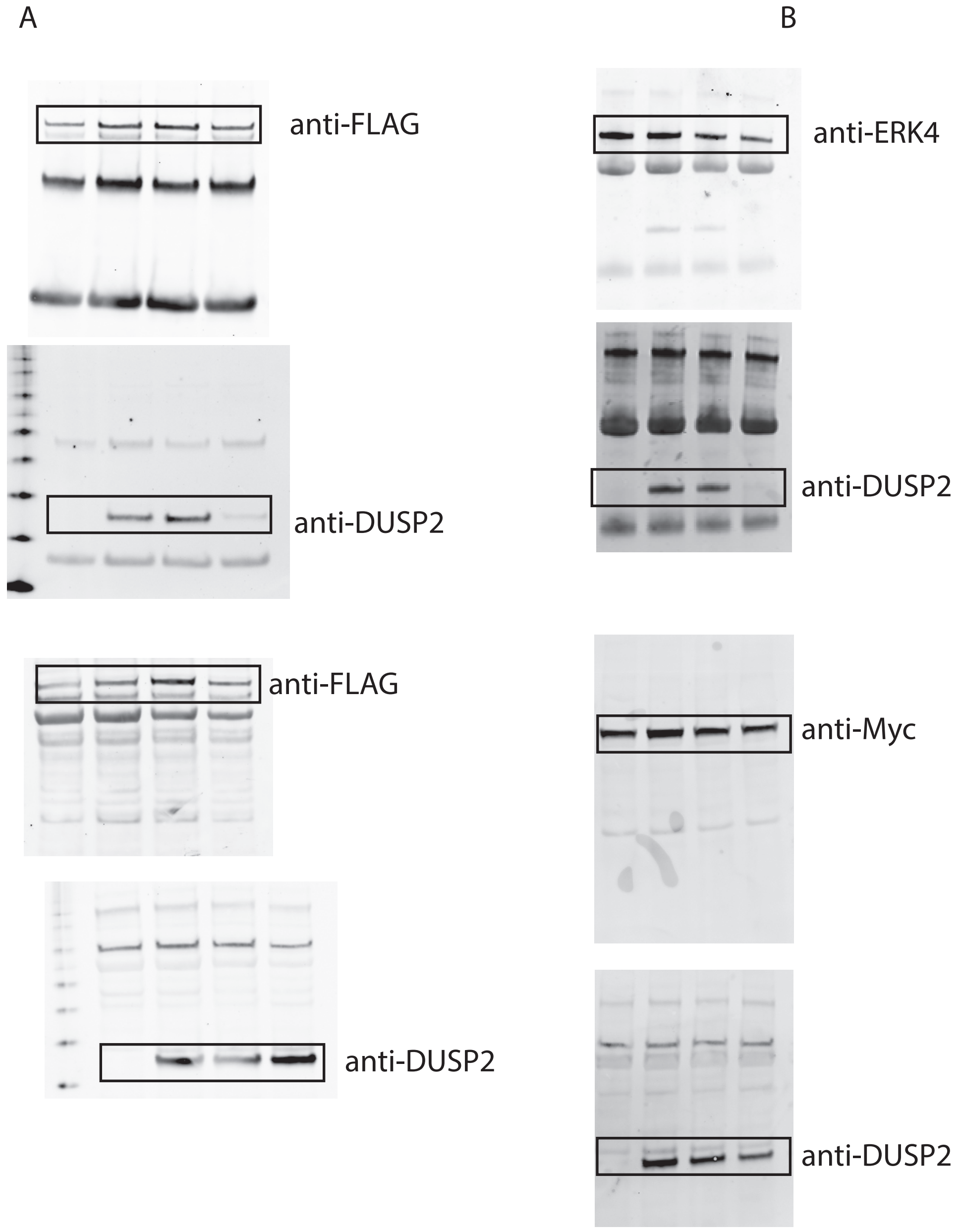
A
B
anti-FLAG
anti-DUSP2
anti-FLAG
anti-DUSP2
anti-ERK4
anti-DUSP2
anti-Myc
anti-DUSP2

Figure 7A

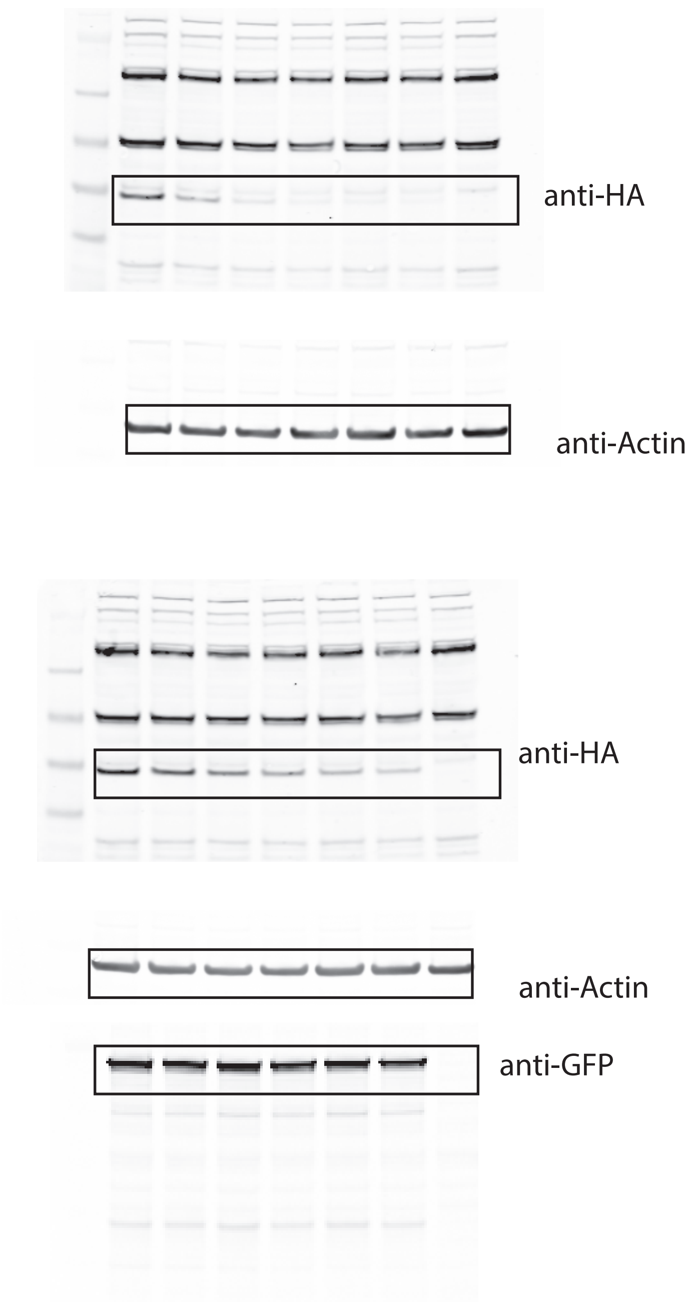

Figure 7B

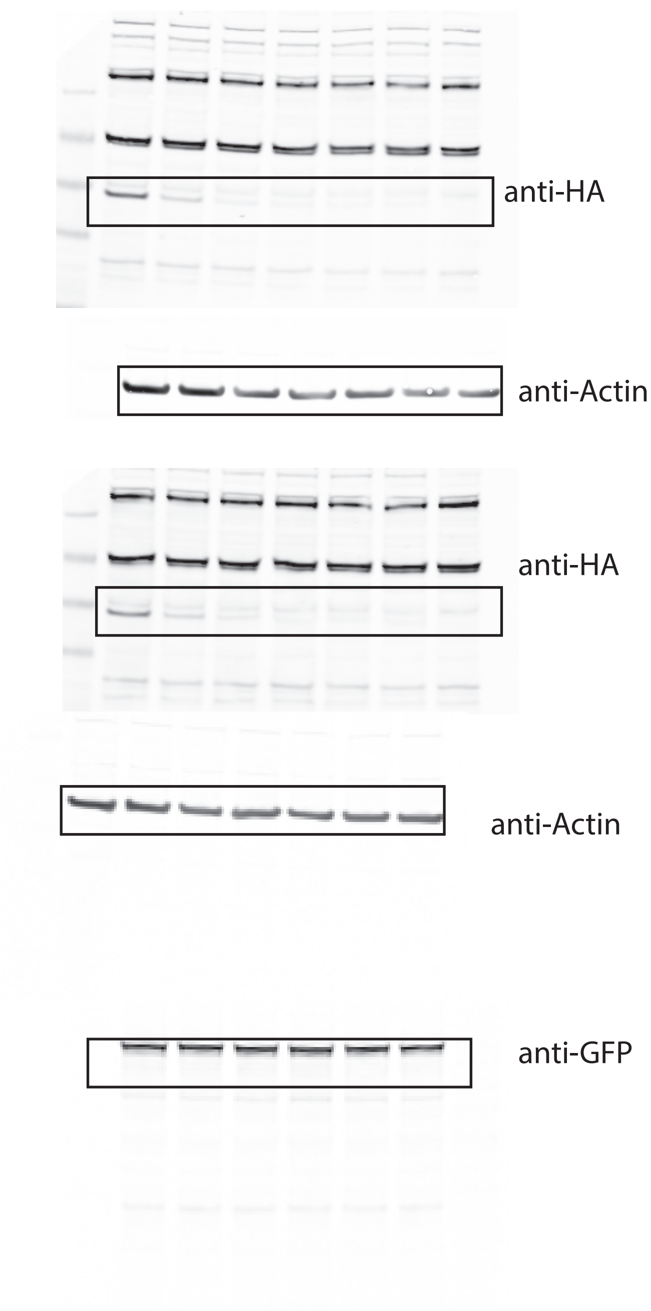

Figure 8A

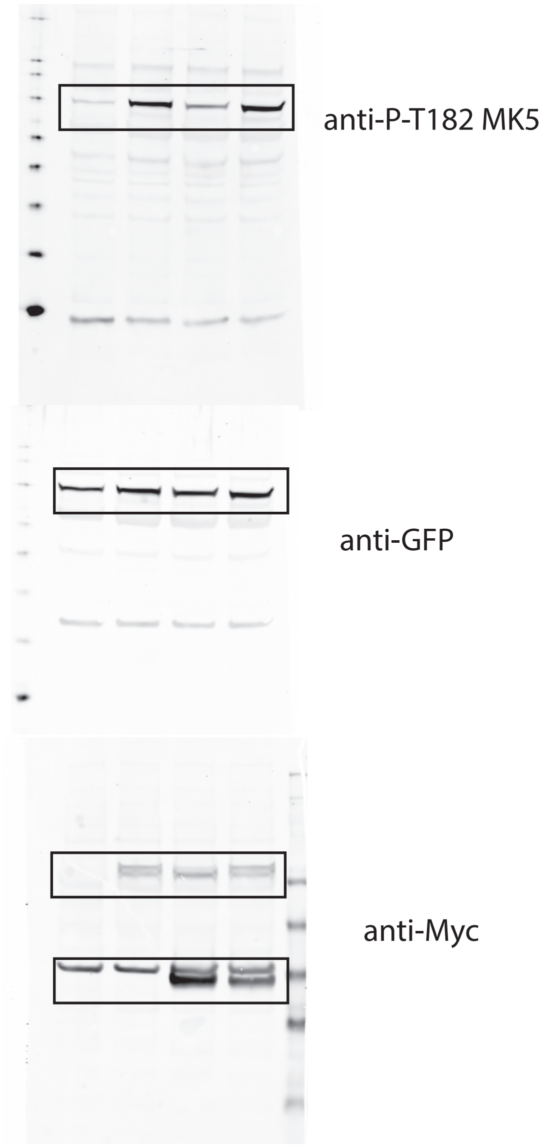

Figure 8C

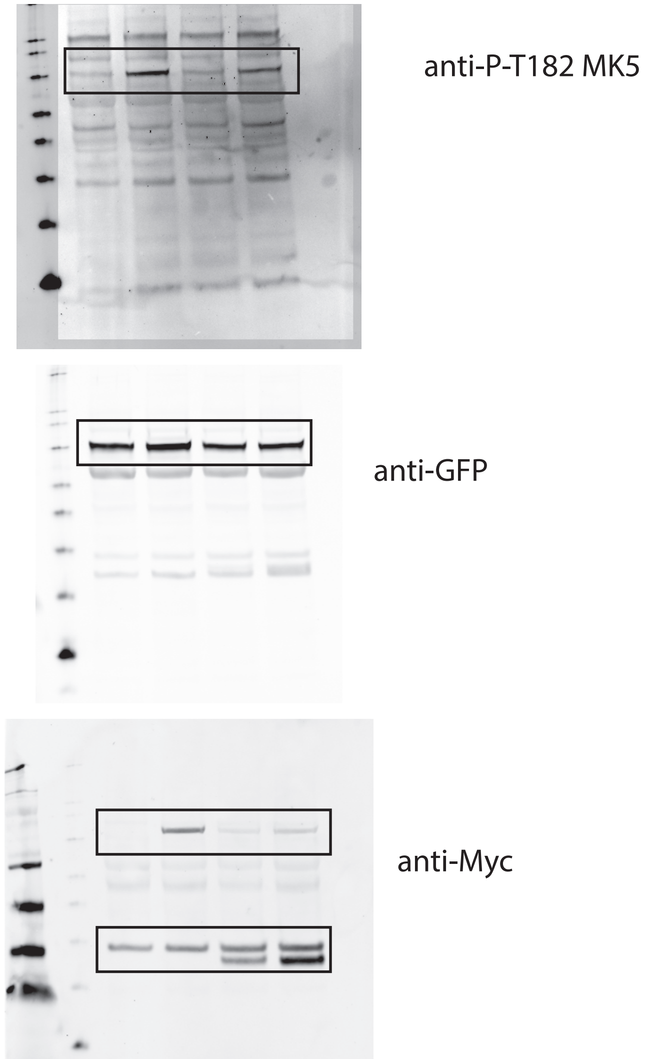

Supplement: Supplementary Information [file srep43471-s1.pdf]
